# Supplementary figures and images for: Hepatic Artery Delineation on Ultrasound Volumes Comparing B-Flow and Color Doppler for Postoperative Monitoring of Pediatric Liver Transplants
Source: Diagnostics (Basel). 2024 Mar 14;14(6):617. doi: 10.3390/diagnostics14060617 (PMC10968933; doi:10.3390/diagnostics14060617)

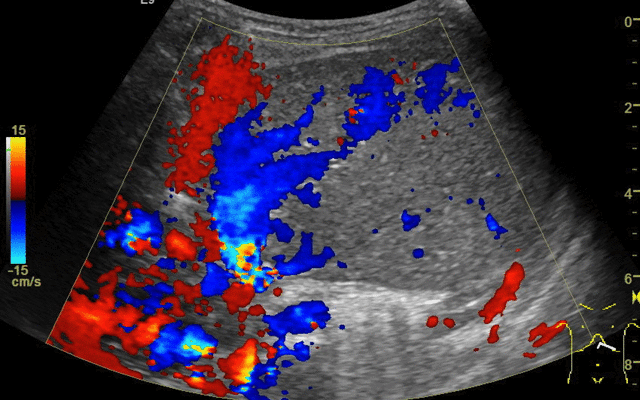

Supplement: Supplementary file 1 [file diagnostics-14-00617-s001.zip › Figure S1_Figure 4 Suppl_CD U1.gif]

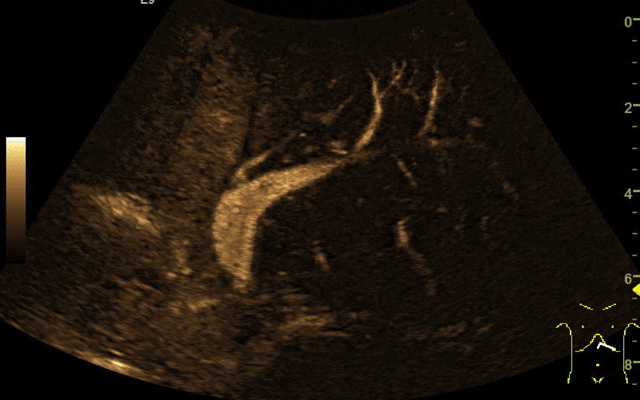

Supplement: Supplementary file 1 [file diagnostics-14-00617-s001.zip › Figure S2_Figure 4 Suppl_B-flow U1.gif]
